# Data fusion of electronic noses and electronic tongues aids in botanical origin identification on imbalanced Codonopsis Radix samples

**DOI:** 10.1038/s41598-022-23857-8

**Published:** 2022-11-09

**Authors:** Shuying Wang, Zhaozhou Lin, Bei Zhang, Jing Du, Wen Li, Zhibin Wang

**Affiliations:** 1 Beijing Zhongyan Tongrentang Medicine R&D Co.Ltd, Beijing, 100079 People’s Republic of China; 2 School of Pharmacy, Lanzhou University, Lanzhou, Gansu 730000 People’s Republic of China

**Keywords:** Analytical chemistry, Cheminformatics, Electrochemistry

## Abstract

Codonopsis Radix (CR) is an edible food and traditional Chinese herb medicine in China. Various varieties of Codonopsis Radix have different tastes. To make the flavor of processed food stable, two kinds of electronic sensory devices, electronic nose and electronic tongue, were used to establish a discrimination model to identify the botanical origin of each sample. The optimal model built on the 88 batches of samples was selected from the models trained with all combination of two pretreatment methods and three classification methods. A comparison were performed on the models trained on the data collected by electronic nose and electronic tongue. The results showed that the model trained on the fused dataset outperformed the models trained separately on the electronic nose data and electronic tongue data. The two preprocessing approaches could improve the prediction performance of all classification methods. Classification and Regression Tree approach performed better than Partial Least Square Discriminant Analysis and Linear Discriminant Analysis in terms of accuracy. But Classification and Regression Tree tends to assign the samples of minority class to the majority class. Meanwhile, Partial Least Square Discriminant Analysis keeps a good balance between the identification requirements of all the two groups of samples. Taking all the results above, the model built using the Partial Least Square Discriminant Analysis method on the fused data after z-score was used to identify the botanical origin of Codonopsis Radix.

## Introduction

Codonopsis Radix is a family of plants used mainly in China and East Asian countries, such as Korea and Japan, to replenish vital energy, or qi^1,2^. The dried roots of *Codonopsis pilosula (Franch.) Nannf, Codonopsis pilosula Nannf. var. modesta (Nannf.) *L. T. Shen and *Codonopsis tangshen Oliv.* are all used as Codonopsis^3^. Traditionally, doctors of Chinese medicine prescribe Codonopsis Radix to strengthen spleen, tonify lung, nourish blood and generate fluid. Besides, Codonopsis Radix is utilized as a nutraceutical component in conventional foods, functional foods, and dietary supplements^4^. The roots are also used in Chinese tonic teas and soups, as well as being roasted with millet, eaten raw, baked, or pickled in miso^5^. However Codonopsis with different sources has different flavor. The roots of *Codonopsis pilosula Nannf. var. modesta (Nannf.)* L. T. Shen have tight skin, thick striate, firm root strips, and sweet-tasting. What’s more, Codonopsis is widely cultivated in Gansu, Shanxi, Shaanxi, Hubei, Sichuan, Jilin province of China, which further diversifies its flavor^6^. Thus, it is necessary to figure out the botanical origin of Codonopsis to keep the taste of foods stable.

The majority of botanical origin identification currently relies on experiential detection, or used in conjunction with laboratory physicochemical detection^7^. However, the subjectivity and insufficient quantitative characterization limit the spread of experiential methods. Although the thin layer chromatography, liquid chromatography, and other methods of laboratory physicochemical detection are frequently used to identify the botanical origin of traditional Chinese medicine (TCM), these methods often come with complex pre-processing processes and protracted detection times^8^.

Thanks to the advancements in sensor manufacturing, it is possible to identify the botanical origin of foods using electronic sensory instruments such as electronic nose and electronic tongue^9–13^. Electronic nose is also known as odor fingerprint technology^14,15^. It functions by simulating the human nose's ability to detect, evaluate, and assess scents. The advantages of simple, fast, and easy pretreatment make it useful for rapid evaluation of complex volatile gas mixtures. The electronic tongue, also known as taste fingerprint technology, is a multi-sensor detection system that analyzes and identifies "taste" by simulating human taste organs^16^. It can accurately and specifically identify the taste, namely sour, bitter, salty, fresh, astringent, and sweet. All the aforementioned electronic sensory equipment have been reported to identify the geographical origin, source, grade of honey^16^, Kiwifruit^17^, Pseudostellariae Radix^18,19^ and Fritillariae Cirrhosae Bulbus^20^. But it remains unclear whether data fusion can improve the classification performance of common classification methods on imbalanced data, such as the botanical origin of Codonopsis Radix.

Upsampling or downsampling the minority or majority class are two techniques to handle imbalanced data for a classification problem. But these two types of methods are unfriendly to the majority of analysts, since they all need some programming skills. In this work, both electronic tongue and nose techniques were employed to gather sensory information about the smell and taste of the Codonopsis samples. To accurately identify the botanical origin of Codonopsis Radix, three classification methods were separately trained on the individual data and the fused one. Data preprocessing method were also employed to eliminate the negative influence cause by data magnitude. The optimal model built on the e-tongue data performs comparable to that on the e-nose data. The results also showed that combining the two different sensors increased the prediction performance on imbalanced data. Even so, there is still much work to be done before a model is ready for usage in industry.

## Materials and methods

### Codonopsis Radix samples

The samples of Codonopsis Radix were gathered from a number of cities in China such as Longnan (Gansu Province), Dingxi (Gansu Province), Changzhi (Shanxi) provinces. A total of 88 samples were dried, crushed and then sifted using a 60 mesh screen. The original plants of the samples were identified by Prof. Hu Fangdi from Lanzhou University. The detailed information of each Codonopsis Radix sample were shown in Supplement Table 1. The experiments for this study were conducted in conformity with the pertinent guidelines and regulations established by the Ministry of Agriculture and Rural Affairs of the People's Republic of China.

### Electronic nose

The PEN3 Electronic Nose system (Airsense Analytics GmBH, Schwerin, Germany) was used in this investigation to detect the smell of the sample. The E-nose system comprise a sampling device, a sensor array and a system software for data acquisition and storage. The sensor array, which is the most important component, is made up of 10 MOS sensors, each of which is sensitive to particular volatile substances (Table 1). The article^17^ defines the primary applications and detection limitations of the 10 MOS sensors.

**Table 1:** Characteristics of MOS sensors utilized in PEN3 electronic nose.

Sensor number	Sensor name	Sensitive substance
S1	W1C	Aromatic constituents, benzene
S2	W5S	Nitroxides
S3	W3C	Ammonia, aromatic ingredients
S4	W6S	Hydrides
S5	W5C	Alkane aromatics
S6	W1S	Short-chain alkanes such as methane
S7	W1W	Inorganic sulfides
S8	W2S	Alcohols, ethers, aldehydes, ketones
S9	W2W	Aromatic component, organic sulfide
S10	W3S	Alkanes, long-chain alkanes

### Electronic tongue

Electronic tongue (e-tongue) measurements were performed with Taste-Sensing System SA 402B (Intelligent Sensor Technology Co., Ltd., Atsugi, Japan), which was designed for the determination of taste of liquid samples. Similar to e-noses, signals from nonspecific sensors were used to mimic the functions of human taste receptors. The five basic flavors of sour, sweet, bitter, salty, and fresh as well as astringent were measured through detecting the changes of membrane potential caused by electrostatic or hydrophobic interaction between flavor substance and artificial lipid membrane. The sensor set includes six sensors, namely AAE, CT0, C00, AE1, CA0, GL1, which stand for umami, saltiness, bitterness, astringency, sourness, and sweetness, respectively^21^. Table 2 details the relationship between the sensors of SA 402B and their physiological meaning.

**Table 2:** Sensors of SA 402B electronic tongue and typical food substances.

Sensor name	Evaluable taste	
	Basic flavor (relative value)	Aftertaste (CPA value)
Umami sensor (AAE)	Umami (caused by amino acids or nucleic acids)	Umami richness (sustainable perceived umami)
Saltiness sensor (CT0)	saltiness (caused by inorganic salts)	None
Sourness sensor (CA0)	Sourness (caused by acetic acid, citric acid, tartaric acid, etc.)	None
Bitterness Sensor (C00)	Bitterness (caused by bitter substances, it is perceived to be richness at low concentrations)	Bitter aftertaste (the bitterness of general foods such as beer and coffee)
Astringency sensor (AE1)	Astringency (caused by astringent substances, it is perceived as a pungent aftertaste at low concentrations)	Astringent aftertaste (the astringent taste of tea, red wine, etc.)
Sweetness sensor (GL1)	Sweetness (caused by sugar or sugar alcohols)	None

### Electronic nose detection

For the method, 3.0 g of each sample was used and put in sample chamber before being tested with the PEN3 electronic nose. Prior to measurement, a sensor check was done to make sure the sensors were operating within the proper voltage range. Each sample was then incubated at 30 ℃ for 60 min in order to reach headspace equilibrium. To normalize the sensor signal, the gas chamber was first cleaned with gas filtered by active charcoal. After each injection and data collection, the sensor self-cleaning time was extended to 120 s to re-establish a stable instrument baseline. The operation parameters were set as following: Sample interval [s]: 1.0; Flush time [s]: 80.0; Zero point trim time: 5.0; Presampling time [s]: 5.0; Measurement time: 80.0; Chamber flow (mL/min): 400; Initial injection flow: 400.

To reduce the sampling error, each sample was measured in triplicate. The data collected at 70 s of the profile were used for the statistical analysis to ensure that the electronic nose reached the adsorption equilibrium. Finally, each sample was represented by a 10-bit vector.

### Electronic tongue detection

For the measurements, 100 mL of each sample filtrate was utilized. The electrodes were conditioned in accordance with the developers' instructions before commencing the experiment. In the beginning, it was necessary to perform a sensor check to make sure all the sensors were operating within the proper voltage range. Every sample measurement began with a cleaning procedure: the sensors were washed for 90 s with the positive and negative cleaning solutions, and then for 120 s with a standard cleaning solution. By measuring the potential of the reference solution after cleaning, the stability of the lipid membrane potential was recorded (Vr). When the sensor response was stable (deviation less than 0.5 mV) during the measurement, the sample solution was examined for 30 s (Vs). The sensor output for taste was formed by the difference between Vr and Vs. After a quick cleaning procedure, the membrane potential was measured once more in standard solution to assess the samples' aftertaste, or CPA (change in membrane potential owing to adsorption). The five fundamental taste signals were measured four times, with the final three results typically being used to guarantee data stability. For the sweetness, one more time was measured.

In addition to the five fundamental taste, three aftertastes of umami, bitterness, and astringency were also recorded. As a result, in a duplication, 9 values will be generated for each sample.

### Data analysis

For the e-nose data, the vectors of duplicate measurements were averaged to reduce measurement variance. Meanwhile, the vector of the last three observations was averaged for each sample of the e-tongue data.

Linear Discriminant Analysis (LDA), or discriminant function analysis is a generalization of Fisher's linear discriminant, a method used in statistics and other fields, to find a linear combination of features that characterizes or separates two or more classes of objects^22^. For the plain LDA method, there is no parameter that need to be optimized, LDA models were directly developed to classify the botanical origin of Codonopsis.

Classification and Regression Trees (CART) is a common classification algorithm that is required to build a decision tree on the basis of Gini’s impurity index. Specifically, the CART algorithm split the nodes into subnodes by searching for the best homogeneity for the subnodes with the help of the Gini index^23^. By default, the decision tree model is allowed to grow to its full depth, which will run the risk of overfitting the training data. In order to prevent this from happening, the decision tree must be pruned. In this study, ten-fold inner cross-validation was used to find the best pruning level.

Partial Least Square-Discriminant Analysis (PLS-DA) is a versatile algorithm commonly used for predictive and descriptive modelling. Over the past two decades, PLS-DA has demonstrated great success in modelling high-dimensional datasets^24^. Despite this, success can still be achieved in low- to middle-dimensional datasets, provided collinearity exists. There are several parameters needs to be optimized before reaching reliable and valid outcomes. One of the most important parameter is the number of latent variables. To provide a trustworthy estimation on the impact of the number of latent variables, eight-fold cross-validation was used.

For binary classification, evaluation metrics including accuracy, precision, sensitivity, specificity, and F1 score are frequently utilized^25^. However, classification accuracy fails for imbalanced class distributions. Thus, the error rate redefined as Eq. (1) was used.

1 $${\text{error rate }} = { 1} - {\text{ mean}}\left( {{\text{sensitivity}}} \right)$$

All the calculation was performed in MATLAB with classification toolbox (Ver. 6.0) developed by Milano Chemometrics and QSAR Research Group^26^.

## Results

### Identification the botanical origins of Codonopsis with electronic nose

**Figure 1:**
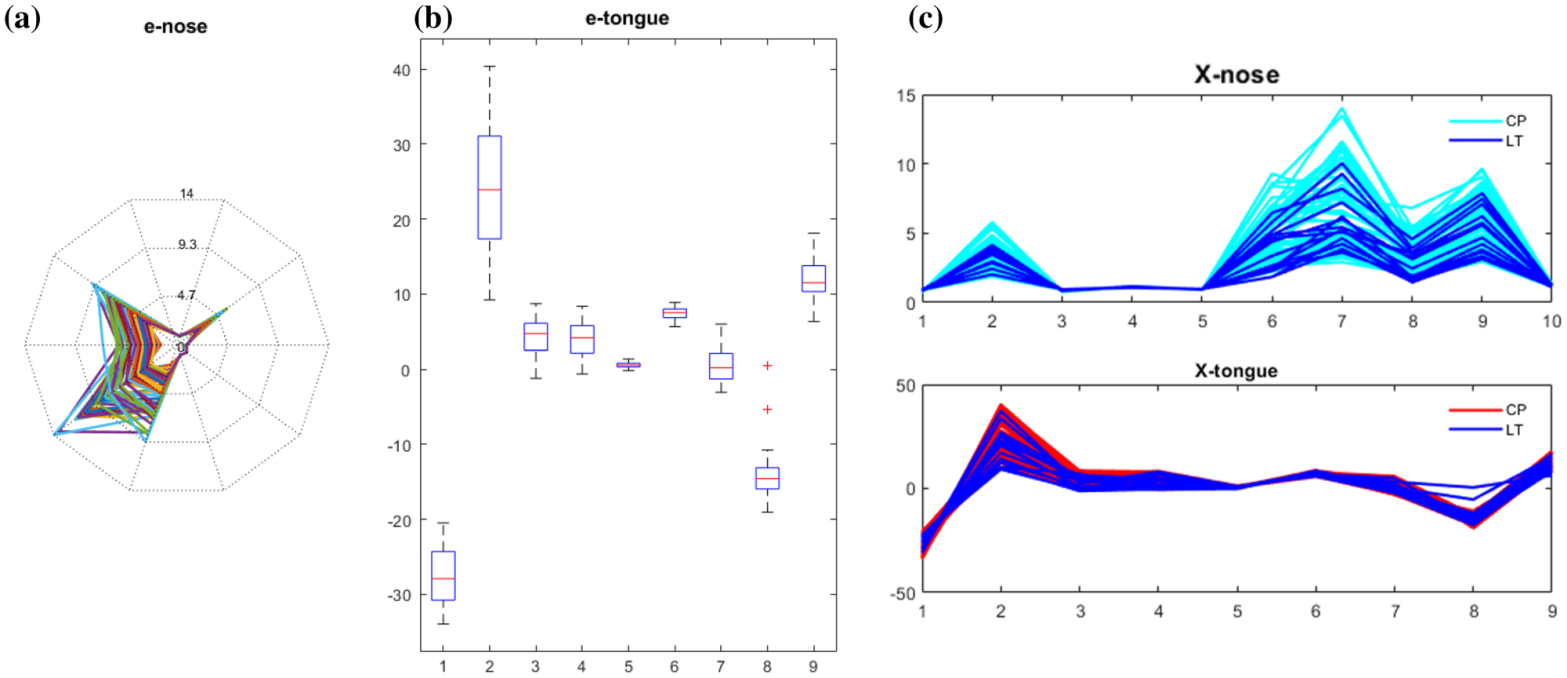
An overview of raw data before variable preprocessing: (**a**) the magnitude and range of different sensors of e-nose; (**b**) the magnitude and range of different sensors of e-tongue; (**c**) the overlay plot of 88 samples.

**Figure 2:**
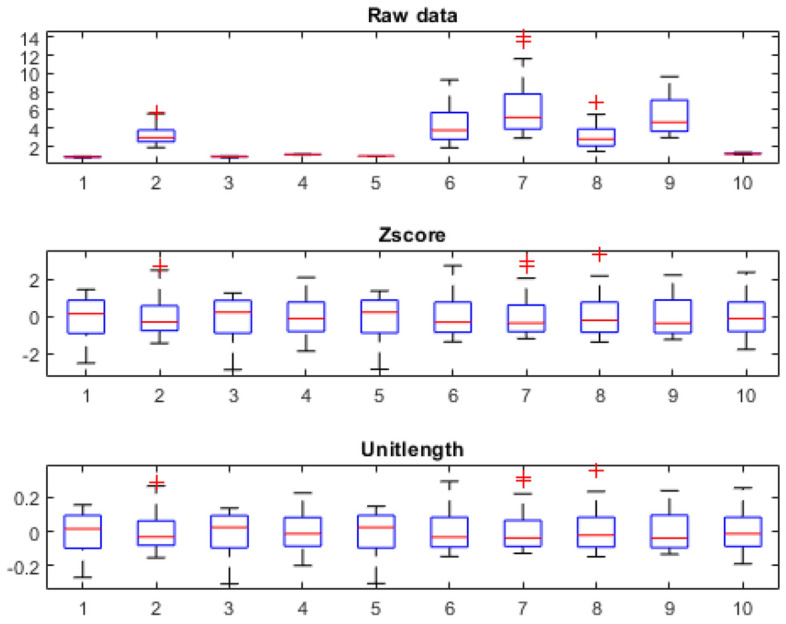
Data distributions after variable preprocessing.

Figure 1 displays the magnitude and range of different sensors of e-nose (a) and e-tongue (b). The response values of both techniques range from a few tenths to tens. Thus, pre-processing techniques must be utilized to reduce the negative effect brought on by the magnitude of various sensors. Scaling to unit length and Z-score Normalization are two commonly used methods in the literature. The aim of scaling to unit length is to scale the components of a feature vector such that the complete vector has length one. This means dividing each component by the Euclidean length of the vector (Unitlength). Standardization, *a.k.a* autoscaling, makes the values of each feature in the data have zero mean and unit variance (Z-score). Figure 2 shows the distribution of data preprocessed by the above two methods. Both preprocessing method align the responses of each sensor to the same level. But the magnitude of data preprocessed by Z-score is ten times larger than that of Unitlength.

From the overlay plot in Fig. 1c, it can be observed that it is hard to clearly distinguish the two group of samples on any sensor of E-nose and E-tongue. Thus, after data pre-processing, three binary classification models were built using CART, LDA and PLS-DA, separately. The results of the optimal model are showed in Table 3. All the metrics were estimated by five-fold cross-validation. For CART in the classification toolbox, the best pruning level was determined automatically by an inner ten-fold cross-validation for each train set in the five-fold cross-validation. But the best pruning level of each train set was not output in the final results. While the number of latent variables in the PLSDA model was optimized by an independent eight-fold cross-validation. The extra cross-validation was used to alleviate the concerns about the potential overly optimistic estimation of the prediction performance.

**Table 3:** The cross validation results of botanical origin identification based on electronic nose.

Methods	Error rate (%)			Accuracy (%)		
	Raw	Autoscaling	Unitlength	Raw	Autoscaling	Unitlength
CART	37	31	31	86	89	89
LDA	33	33	33	85	85	85
PLSDA (n.lvs)	30 (5)	28 (2)	28 (2)	81 (5)	76 (2)	76 (2)

n.lvs in the parenthesis denotes the number of latent variables optimized by eight-fold cross-validation.

**Figure 3:**
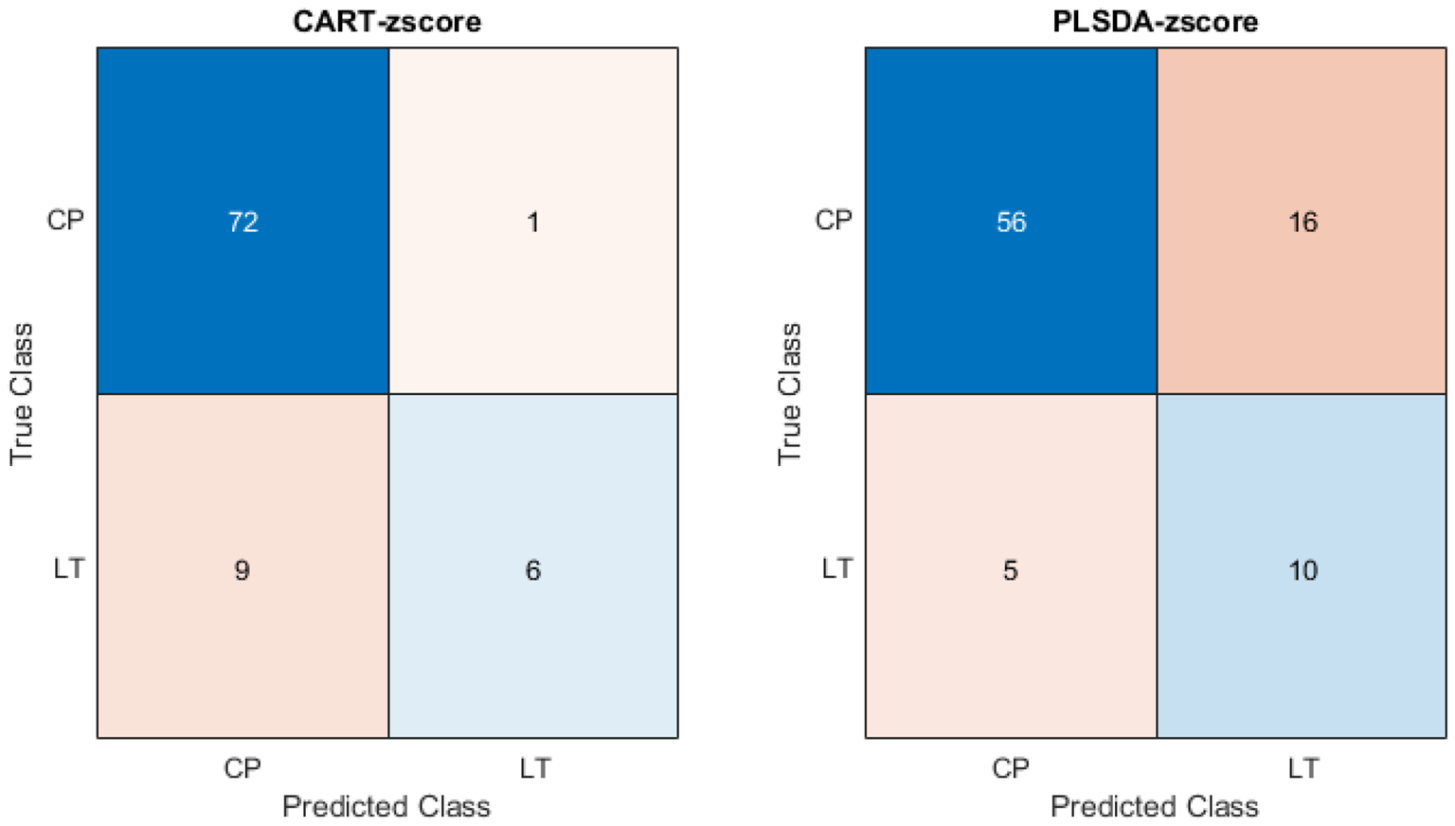
The confusion matrix of CART and PLSDA on the electronic nose data.

The error rate in this table illustrate that the prediction performance can be improved obviously following pretreatment. The same conclusion can be obtained in terms of accuracy. Based on error rate, the comparison of the models' performances reveals that PLSDA performs better than LDA and CART. However, with respect to their accuracy, the conclusion is reversed. From the confusion matrix (Fig. 3), it can be seen that CART tends to assign the sample to *Codonopsis pilosula (Franch.) Nannf* (the majority class), while PLSDA model tends to classify the sample as *Codonopsis pilosula Nannf. var. modesta (Nannf.) L. T. Shen* (the minority class). In fact, the sample size of the two groups is unbalanced. If all the samples were classified as *Codonopsis pilosula (Franch.) Nannf*, the model accuracy rate could reach 83%.

### Identification the botanical origin of Codonopsis Radix with electronic tongue

Similar to the electronic nose data, data preprocessing was executed on the electronic tongue data to eliminate the influence caused by data range across sensors. Undoubtedly, the prediction performance of these models can be improved more or less after pretreatment. As is shown in Table 4, no method is always performed better than the others. The LDA model outperformed CART model in terms of both error rate and accuracy. Compared with LDA, the error rate of PLSDA was reduced significantly after preprocessing. While the accuracy of PLSDA model on the z-score data exhibited a different trend.

**Table 4:** The cross validation results of botanical origin identification based on electronic tongue.

Methods	Error rate (%)			Accuracy(%)		
	Raw	Autoscaling	Unitlength	Raw	Autoscaling	Unitlength
CART	47	37	47	80	86	80
LDA	33	33	33	84	85	84
PLSDA	38	28	16	64	76	88

To appreciate how these diverse results arose, the prediction details were summarized in Table 5. There is a huge difference between the number of samples not assigned to any group predicted by PLSDA and LDA. But the number of samples not assigned could not explain the performance of PLSDA on the Z-score data. Taking the results presented in Table 3 into consideration, the reason can be attributed to the preference of the two classification methods.

**Table 5:** The classification results of LDA and PLSDA on electronic tongue data.

Methods		Real	Predicted		
			CP	LT	Not assigned
LDA	Raw	CP	68	5	0
		LT	9	6	0
	auto	CP	69	4	0
		LT	9	6	0
	unil	CP	68	5	0
		LT	9	6	0
PLSDA	Raw(2)	CP	31	17	25
		LT	4	6	5
	auto(5)	CP	56	16	1
		LT	5	10	0
	unil(5)	CP	61	7	5
		LT	3	11	1

**auto** stands for Autoscaling; **unil** stands for Unitlength; **CP** is short for *Codonopsis pilosula (Franch.) Nannf*; **LT** is short for *Codonopsis pilosula Nannf. var. modesta (Nannf.) L. T. Shen; the number in the parenthesis denotes the number of latent variables optimized by eight-fold cross-validation*.

### Identification the botanical origin of Codonopsis Radix with electronic nose and electronic tongue

The results obtained on the electronic tongue data and electronic nose data both demonstrated that preprocessing could improve the prediction performance of classification methods. However, since the number of samples not assigned to the data preprocessed by Unitlength is larger than that of z-score in Table 5, data fusion was directly executed on the data preprocessed by z-score.

From the raw data extracted from the electronic nose (X1) and electronic tongue (X2), it can be observed that there are few variables extracted (X1 has 10 variables and X2 has 9 variables). Thus, the two datasets were augmented first to pursue possible improvement in the classification model. The hyperparameters of CART, LDA, and PLSDA were tuned using cross-validation. The final result was presented in Table 6.

**Table 6:** The classification performance of CART, LDA and PLSDA on data fused with two different strategies.

Methods		Real	Predicted			Error rate (%)	Accuracy rate (%)
			CP	LT	Not assigned		
CART	Augment	CP	72	1	0	37	86
		LT	11	4	0		
	PCA	CP	68	5	0	50	78
		LT	14	1	0		
LDA	Augment	CP	64	9	0	29	82
		LT	7	8	0		
	PCA	CP	69	4	0	49	80
		LT	14	1	0		
PLSDA	Augment(12)	CP	62	11	0	14	85
		LT	2	13	0		
	PCA(1)	CP	46	27	0	42	61
		LT	7	8	0		

**CP** is short for *Codonopsis pilosula (Franch.) Nannf*; **LT** is short for *Codonopsis pilosula Nannf. var. modesta (Nannf. )* *L. T. Shen; the number in the parenthesis denotes the number of latent variables optimized by eight-fold cross-validation*.

Taking the cross-validation accuracy as an index, the three classification models rank as follows: CART > PLSDA > LDA. While ranked with respect to the error rate of cross-validation, the three models are ordered in the opposite sequence. In fact, it is evident from the confusion matrix that CART outperforms PLSDA by a small margin, but with a stronger trend to classify the sample of *Codonopsis pilosula Nannf. var. modesta (Nannf.) L. T. Shen* as *Codonopsis pilosula (Franch.) Nannf.* While, the PLSDA model not only maintains high prediction accuracy but also strikes a balance between the need to correctly distinguish the two groups of Codonopsis Radix. Therefore, based on the aforementioned findings, PLSDA should be chosen as the final modeling approach on the augmented data.

Comparing the results of PLSDA model in Tables 3, 4 and 6, it can be observed that augmenting the electronic nose data with the electronic tongue data could enhance the classification performance. Previous studies^27^ have demonstrated that PCA is an effective data presentation learning method and the features learned could meet the general requirements of mid-level data fusion methods. But it remains unclear whether PCA works for the data when the number of samples is larger than the number of variables. Thus, principle components (PCs) were learned from the electronic nose data and the electronic tongue data separately. Then, the PCs were used in conjunction to train a classification model.

The number of PCs was chosen as the PCs whose cumulating contribution rate was greater than 90%. For the e-tongue data, the first three PCs explained 93.43% of total variance. Meanwhile, the first two PCs explained 98.57% of total variance of e-nose data. The PCs were merged into a matrix **Xpc** of size (88, 5). Then, the CART, LDA, and PLSDA models were trained on the feature matrix **Xpc**.

The variance of the explained of the first three principal components of e-tongue was 93.43%, and the cumulative contribution of the first two principal components of e-nose was 98.57%. The new matrix **Xpc **(88*5) was obtained by combining the principal component scores of the two types of data, and then classification models were established with CART, LDA and PLSDA respectively. The results were shown in Table 6.

As shown in Table 6, the models built on **Xpc** performed worse than their corresponding model on the raw data. Both CART and LDA still prone to identify the samples as *Codonopsis pilosula (Franch.) Nannf.* Although the performance of PLSDA was worse than CART and LDA, it reserve the ability to discern sample of *Codonopsis pilosula Nannf. var. modesta (Nannf.) L. T. Shen*. Even though, there are no samples not assigned to any group. In short, the data fusion strategy based on PCA is not suitable for our data. However, whether this conclusion can be extended to the data that the number of samples is larger than the number of variables remains to be verified.

## Discussion

The results of models trained with different classification methods before and after data preprocessing showed that data pretreatment could enhance the classification performance of models built on the data of both e-nose and e-tongue. Although the classification methods used in this study are diverse in their theoretical background, none performed better than the other two in all metrics. From the boxplot in Fig. 2, it was observed that both z-score and Unitlength aligned the responses of different sensors to the same level. By doing so, the contribution of sensors with slower responses will be amplified. That might explain the improvement of data pretreatment on classification models.

Generally, PLSDA is generally used in cases with more variables than samples^28^. The PLSDA methods were used in this study since collinearity problem existed in e-nose and e-tongue data. Collinearity introduces the risk of over-fitting when using the classification method based on the inverse of the Matrix. When applying LDA to e-tongue and e-nose separately and fused data, the prediction error rate and accuracy of the resubmission test are much better than that of cross validation.

Although merging the data of multi-sensors is simple, it is an effective data fusion method^29^. The results obtained in this study demonstrated that augmenting the e-tongue data with e-nose data could improve the model’s ability to distinguish the samples of *Codonopsis pilosula Nannf. var. modesta (Nannf. ) L. T. Shen* from *Codonopsis pilosula (Franch.) Nannf*. However, in the Codonopsis Radix dataset, the majority class is much larger than the minority class. Both CART and LDA failed to train an impartial model; they all tended to assign samples to the majority class. While PLSDA keeps a good balance between the two classes. Further research is required to determine whether PLSDA can fulfill the requirement of minority class classification for other imbalanced data.

## Conclusions

In this study, 88 batches of Codonopsis Radix sample were measured by electronic nose and electronic tongue. The collected data was used separately or in conjunction to create a classification model capable of correctly determine the botanical origin of each sample. The results showed that by merging the electronic nose data and electronic tongue data into a single data set could assist the PLSDA method in building a usable classification model even if the dataset is highly imbalanced. Specifically, the error rate reduced to 14% from 28% on e-nose or 16% on e-tongue. Moreover, it was found that both pretreatment methods used in this study could improve the prediction performance of the models. In other words, the magnitude and range of electronic sensors have a great influence on the prediction ability of the model. But data fusion based on features extract by PCA failed to further improve the prediction performance. More effort should be made to make it clear what the cause of this phenomenon is.

## Supplementary Information

Supplementary Table 1.

## Data Availability

The raw data generated during the current study are not publicly available due to privacy restrictions, but the derived data supporting the findings of this study are available from the corresponding author on reasonable request. Sample collection: The authors declare that they have a license to collect two varieties of Codonopsis Radix (*Codonopsis pilosula (Franch.) Nannf, Codonopsis pilosula Nannf. var. modesta (Nannf.) *L. T. Shen). The authors declare that they comply with the IUCN Policy Statement on Research Involving Species at Risk of Extinction and the Convention on the Trade in Endangered Species of Wild Fauna and Flora.

## References

[1] Gao S-M, Liu J-S, Wang M, Cao T-T, Qi Y-D, Zhang B-G, Sun X-B, Liu H-T, Xiao P-G (2018). Traditional uses, phytochemistry, pharmacology and toxicology of Codonopsis: A review. J. Ethnopharmacol..

[2] Bai R, Wang Y, Fan J, Zhang J, Li W, Zhang Y, Hu F (2022). Intra-regional classification of Codonopsis Radix produced in Gansu province (China) by multi-elemental analysis and chemometric tools. Sci. Rep..

[3] Chinese Pharmacopoeia Commission, *Chinese Pharmacopoeia* 2020. Vol. **I**, 293 (2020)

[4] Wang Y, Zhang J-J, Wang Z-X, Cui F, Zhang Q-N, Song P-P, Li B, Tang Z-S, Hu F-D, Shi X-F (2022). Characterization of chemical composition variations in raw and processed Codonopsis Radix by integrating metabolomics and glycomics based on multiple chromatography-mass spectrometry technology. J. Sep. Sci..

[5] Zou Y-F, Zhang Y-Y, Paulsen BS, Fu Y-P, Huang C, Feng B, Li L-X, Chen X-F, Jia R-Y, Song X, He C-L, Yin L-Z, Ye G, Liang X-X, Lv C, Yin Z-Q (2020). Prospects of *Codonopsis pilosula* polysaccharides: Structural features and bioactivities diversity. Trends Food Sci. Tech..

[6] Liu X, Zhao H, Liu Y, Cheng R, Liu X, Wang X, Jiao J (2010). Study and Comparison on HPLC finger printing of radix codonopsitis from different habitats. J. Shanxi Coll. Trad. Ch. Med..

[7] Gao H, Wang Z, Li Y, Qian Z (2011). Overview of the quality standard research of traditional Chinese medicine. Front. Med..

[8] Xie P-S, Leung AY (2009). Understanding the traditional aspect of Chinese medicine in order to achieve meaningful quality control of Chinese materia medica. J. Chromatogr. A.

[9] Xu M, Yang S-L, Peng W, Liu Y-J, Xie D-S, Li X-Y, Wu C-J (2015). A novel method for the discrimination of semen arecae and its processed products by using computer vision, electronic nose, and electronic tongue. Evid-Based Compl. Alt..

[10] Di Rosa AR, Leone F, Cheli F, Chiofalo V (2017). Fusion of electronic nose, electronic tongue and computer vision for animal source food authentication and quality assessment—a review. J. Food Eng..

[11] Aouadi B, Zaukuu J-LZ, Vitális F, Bodor Z, Fehér O, Gillay Z, Bazar G, Kovacs Z (2020). Historical evolution and food control achievements of near infrared spectroscopy, electronic nose, and electronic tongue—critical overview. Sensors.

[12] Tan J, Xu J (2020). Applications of electronic nose (e-nose) and electronic tongue (e-tongue) in food quality-related properties determination: A review. Artif. Intell. Agr..

[13] Vilela A, Bacelar E, Pinto T, Anjos R, Correia E, Gonçalves B, Cosme F (2019). Beverage and food fragrance biotechnology, novel applications, sensory and sensor techniques: An overview. Foods.

[14] Rottiers H, Tzompa Sosa DA, Van de Vyver L, Hinneh M, Everaert H, De Wever J, Messens K, Dewettinck K (2019). Discrimination of cocoa liquors based on their odor fingerprint: A fast GC electronic nose suitability study. Food Anal. Methods.

[15] Bonah E, Huang X, Aheto JH, Osae R (2020). Application of electronic nose as a non-invasive technique for odor fingerprinting and detection of bacterial foodborne pathogens: A review. J. Food Sci. Tech..

[16] Jiang H, Zhang M, Bhandari B, Adhikari B (2018). Application of electronic tongue for fresh foods quality evaluation: A review. Food Rev. Int..

[17] Du D, Wang J, Wang B, Zhu L, Hong X (2019). Ripeness prediction of postharvest kiwifruit using a MOS e-nose combined with chemometrics. Sensors.

[18] Huang T, Sun Y, Guo Y, Wang W, He T, Cao J (2022). Application of HS-SPME-GC-MS combined with electronic nose technology in the odor recognition of pseudostellariae radix. Nat. Prod. Res..

[19] Huang T-H (2020). Identification of Pseudostellaria radix from different producing regions and habitat processing methods based on electronic nose technology. Ch. Phar. J..

[20] Yang S, Xie S, Xu M, Zhang C, Wu N, Yang J, Zhang L, Zhang D, Jiang Y, Wu C (2015). A novel method for rapid discrimination of bulbus of Fritillaria by using electronic nose and electronic tongue technology. Anal. Methods.

[21] Matsuo Y, Akita K, Taguchi H, Fujii S, Yoshie-Stark Y, Araki T (2022). Utilization and evaluation of *Citrus natsudaidai* peel waste as a source of natural food additives. Food Chem..

[22] Behmann J, Mahlein A-K, Rumpf T, Römer C, Plümer L (2015). A review of advanced machine learning methods for the detection of biotic stress in precision crop protection. Precis. Agric..

[23] Singh S, Gupta P (2014). Comparative study ID3, cart and C45 decision tree algorithm: A survey. Int. J. Adv. Inf. Sci. Technol..

[24] Westerhuis JA, van Velzen EJ, Hoefsloot HC, Smilde AK (2010). Multivariate paired data analysis: Multilevel PLSDA versus OPLSDA. Metabolomics.

[25] Tharwat A (2020). Classification assessment methods. Appl. Com. Inf..

[26] Ballabio D, Consonni V (2013). Classification tools in chemistry. Part 1: Linear models. PLS-DA. Anal. Methods.

[27] Dai S-Y, Lin Z-Z, Xu B, Wang Y-Q, Shi X-Y, Qiao Y-J, Zhang J-Y (2018). Metabolomics data fusion between near infrared spectroscopy and high-resolution mass spectrometry: A synergetic approach to boost performance or induce confusion. Talanta.

[28] Brereton RG, Lloyd GR (2014). Partial least squares discriminant analysis: Taking the magic away. J. Chemometr..

[29] Calvini R, Pigani L (2022). Toward the development of combined artificial sensing systems for food quality evaluation: A review on the application of data fusion of electronic noses, electronic tongues and electronic eyes. Sensors.

