# Supplementary material for: Data fusion of electronic noses and electronic tongues aids in botanical origin identification on imbalanced Codonopsis Radix samples
Source: Sci Rep. 2022 Nov 9;12:19120. doi: 10.1038/s41598-022-23857-8 (PMC9646742; doi:10.1038/s41598-022-23857-8)
Supplement: Supplementary file 1 — Supplementary Table 1. [file 41598_2022_23857_MOESM1_ESM.docx]

**Supplement Table 1**. The basic information of Codonopsis Radix samples

| No. | Source Plant | Producing Region |
| --- | --- | --- |
| 1 | Codonopsis pilosula Nannf. var. modesta （Nannf. ）L. T. Shen | Longnan, Gansu Province |
| 2 | Codonopsis pilosula Nannf. var. modesta （Nannf. ）L. T. Shen | Longnan, Gansu Province |
| 3 | Codonopsis pilosula Nannf. var. modesta （Nannf. ）L. T. Shen | Longnan, Gansu Province |
| 4 | Codonopsis pilosula Nannf. var. modesta （Nannf. ）L. T. Shen | Weining, Guizhou Province |
| 5 | Codonopsis pilosula (Franch.) Nannf | Dingxi, Gansu Province |
| 6 | Codonopsis pilosula (Franch.) Nannf | Dingxi, Gansu Province |
| 7 | Codonopsis pilosula (Franch.) Nannf | Dingxi, Gansu Province |
| 8 | Codonopsis pilosula (Franch.) Nannf | Dingxi, Gansu Province |
| 9 | Codonopsis pilosula (Franch.) Nannf | Dingxi, Gansu Province |
| 10 | Codonopsis pilosula Nannf. var. modesta （Nannf. ）L. T. Shen | Longnan, Gansu Province |
| 11 | Codonopsis pilosula (Franch.) Nannf | Dingxi, Gansu Province |
| 12 | Codonopsis pilosula (Franch.) Nannf | Dingxi, Gansu Province |
| 13 | Codonopsis pilosula (Franch.) Nannf | Dingxi, Gansu Province |
| 14 | Codonopsis pilosula (Franch.) Nannf | Longnan, Gansu Province |
| 15 | Codonopsis pilosula (Franch.) Nannf | Changzhi, Shanxi provinces |
| 16 | Codonopsis pilosula (Franch.) Nannf | Dingxi, Gansu Province |
| 17 | Codonopsis pilosula (Franch.) Nannf | Dingxi, Gansu Province |
| 18 | Codonopsis pilosula (Franch.) Nannf | Dingxi, Gansu Province |
| 19 | Codonopsis pilosula (Franch.) Nannf | Dingxi, Gansu Province |
| 20 | Codonopsis pilosula (Franch.) Nannf | Dingxi, Gansu Province |
| 21 | Codonopsis pilosula (Franch.) Nannf | Dingxi, Gansu Province |
| 22 | Codonopsis pilosula (Franch.) Nannf | Dingxi, Gansu Province |
| 23 | Codonopsis pilosula Nannf. var. modesta （Nannf. ）L. T. Shen | Longnan, Gansu Province |
| 24 | Codonopsis pilosula (Franch.) Nannf | Dingxi, Gansu Province |
| 25 | Codonopsis pilosula (Franch.) Nannf | Dingxi, Gansu Province |
| 26 | Codonopsis pilosula (Franch.) Nannf | Dingxi, Gansu Province |
| 27 | Codonopsis pilosula (Franch.) Nannf | Dingxi, Gansu Province |
| 28 | Codonopsis pilosula (Franch.) Nannf | Longnan, Gansu Province |
| 29 | Codonopsis pilosula (Franch.) Nannf | Dingxi, Gansu Province |
| 30 | Codonopsis pilosula (Franch.) Nannf | Dingxi, Gansu Province |
| 31 | Codonopsis pilosula (Franch.) Nannf | Dingxi, Gansu Province |
| 32 | Codonopsis pilosula (Franch.) Nannf | Dingxi, Gansu Province |
| 33 | Codonopsis pilosula (Franch.) Nannf | Dingxi, Gansu Province |
| 34 | Codonopsis pilosula Nannf. var. modesta （Nannf. ）L. T. Shen | Enshi, Hubei Province |
| 35 | Codonopsis pilosula (Franch.) Nannf | Dingxi, Gansu Province |
| 36 | Codonopsis pilosula (Franch.) Nannf | Dingxi, Gansu Province |
| 37 | Codonopsis pilosula (Franch.) Nannf | Dingxi, Gansu Province |
| 38 | Codonopsis pilosula (Franch.) Nannf | Longnan, Gansu Province |
| 39 | Codonopsis pilosula (Franch.) Nannf | Longnan, Gansu Province |
| 40 | Codonopsis pilosula (Franch.) Nannf | Daozhen Autonomous County Xuanyang Pharmaceutical Co. , Ltd. |
| 41 | Codonopsis pilosula Nannf. var. modesta （Nannf. ）L. T. Shen | Longnan, Gansu Province |
| 42 | Codonopsis pilosula (Franch.) Nannf | Changzhi, Shanxi provinces |
| 43 | Codonopsis pilosula (Franch.) Nannf | Changzhi, Shanxi provinces |
| 44 | Codonopsis pilosula (Franch.) Nannf | Changzhi, Shanxi provinces |
| 45 | Codonopsis pilosula (Franch.) Nannf | Gannan, Gansu Province |
| 46 | Codonopsis pilosula Nannf. var. modesta （Nannf. ）L. T. Shen | Longnan, Gansu Province |
| 47 | Codonopsis pilosula Nannf. var. modesta （Nannf. ）L. T. Shen | Longnan, Gansu Province |
| 48 | Codonopsis pilosula Nannf. var. modesta （Nannf. ）L. T. Shen | Longnan, Gansu Province |
| 49 | Codonopsis pilosula (Franch.) Nannf | Longnan, Gansu Province |
| 50 | Codonopsis pilosula Nannf. var. modesta （Nannf. ）L. T. Shen | Longnan, Gansu Province |
| 51 | Codonopsis pilosula (Franch.) Nannf | Longnan, Gansu Province |
| 52 | Codonopsis pilosula Nannf. var. modesta （Nannf. ）L. T. Shen | Longnan, Gansu Province |
| 53 | Codonopsis pilosula (Franch.) Nannf | Dingxi, Gansu Province |
| 54 | Codonopsis pilosula (Franch.) Nannf | Dingxi, Gansu Province |
| 55 | Codonopsis pilosula (Franch.) Nannf | Dingxi, Gansu Province |
| 56 | Codonopsis pilosula (Franch.) Nannf | Dingxi, Gansu Province |
| 57 | Codonopsis pilosula (Franch.) Nannf | Dingxi, Gansu Province |
| 58 | Codonopsis pilosula (Franch.) Nannf | Gannan, Gansu Province |
| 59 | Codonopsis pilosula (Franch.) Nannf | Gannan, Gansu Province |
| 60 | Codonopsis pilosula (Franch.) Nannf | Dingxi, Gansu Province |
| 61 | Codonopsis pilosula (Franch.) Nannf | Dingxi, Gansu Province |
| 62 | Codonopsis pilosula (Franch.) Nannf | Dingxi, Gansu Province |
| 63 | Codonopsis pilosula (Franch.) Nannf | Dingxi, Gansu Province |
| 64 | Codonopsis pilosula (Franch.) Nannf | Dingxi, Gansu Province |
| 65 | Codonopsis pilosula (Franch.) Nannf | Dingxi, Gansu Province |
| 66 | Codonopsis pilosula (Franch.) Nannf | Dingxi, Gansu Province |
| 67 | Codonopsis pilosula (Franch.) Nannf | Dingxi, Gansu Province |
| 68 | Codonopsis pilosula (Franch.) Nannf | Dingxi, Gansu Province |
| 69 | Codonopsis pilosula Nannf. var. modesta （Nannf. ）L. T. Shen | Dingxi, Gansu Province |
| 70 | Codonopsis pilosula (Franch.) Nannf | Dingxi, Gansu Province |
| 71 | Codonopsis pilosula (Franch.) Nannf | Dingxi, Gansu Province |
| 72 | Codonopsis pilosula (Franch.) Nannf | Longnan, Gansu Province |
| 73 | Codonopsis pilosula (Franch.) Nannf | Dingxi, Gansu Province |
| 74 | Codonopsis pilosula (Franch.) Nannf | Longnan, Gansu Province |
| 75 | Codonopsis pilosula (Franch.) Nannf | Longnan, Gansu Province |
| 76 | Codonopsis pilosula (Franch.) Nannf | Dingxi, Gansu Province |
| 77 | Codonopsis pilosula (Franch.) Nannf | Dingxi, Gansu Province |
| 78 | Codonopsis pilosula (Franch.) Nannf | Longnan, Gansu Province |
| 79 | Codonopsis pilosula (Franch.) Nannf | Dingxi, Gansu Province |
| 80 | Codonopsis pilosula (Franch.) Nannf | Longnan, Gansu Province |
| 81 | Codonopsis pilosula (Franch.) Nannf | Dingxi, Gansu Province |
| 82 | Codonopsis pilosula (Franch.) Nannf | Dingxi, Gansu Province |
| 83 | Codonopsis pilosula (Franch.) Nannf | Longnan, Gansu Province |
| 84 | Codonopsis pilosula (Franch.) Nannf | Longnan, Gansu Province |
| 85 | Codonopsis pilosula (Franch.) Nannf | Longnan, Gansu Province |
| 86 | Codonopsis pilosula Nannf. var. modesta （Nannf. ）L. T. Shen | Longnan, Gansu Province |
| 87 | Codonopsis pilosula (Franch.) Nannf | Dingxi, Gansu Province |
| 88 | Codonopsis pilosula (Franch.) Nannf | Longnan, Gansu Province |
